# Human-centered design of a smartphone-based self-test for HIV viral load monitoring

**DOI:** 10.1017/cts.2023.686

**Published:** 2023-11-24

**Authors:** Natalia M. Rodriguez, Lara Balian, Cealia Tolliver, Ishita Kataki, Julio Rivera-De Jesus, Jacqueline C. Linnes

**Affiliations:** 1 Department of Public Health, College of Health and Human Sciences, Purdue University, West Lafayette, IN, USA; 2 Weldon School of Biomedical Engineering, College of Engineering, Purdue University, West Lafayette, IN, USA

**Keywords:** HIV viral load, self-testing, human-centered design, smartphone-based diagnostic, technological innovation

## Abstract

**Background/Objective:**

HIV viral load self-testing could enable people living with HIV (PLHIV) to monitor their viral suppression status more easily, potentially facilitating medication adherence and safe behavior decision-making. Smartphone-based viral load testing innovations have the potential to reach resource-limited and vulnerable communities to address inequities in access to HIV care. However, successful development and translation of these tests requires meaningful investigation of end-user contexts and incorporation of those context-specific needs early in the design process. The objective of this study is to engage PLHIV and HIV healthcare providers in human-centered design research to inform key design and implementation considerations for a smartphone-based HIV viral load self-testing device prototype in development.

**Methods:**

Semi-structured in-depth interviews were conducted with PLHIV (*n* = 10) and HIV providers (*n* = 4) in Indiana, a state with suboptimal viral suppression rates and marked disparities in access to HIV care. Interview guides were developed based on contextual investigation and human-centered design frameworks and included a demonstration of the device prototype with feedback-gathering questions.

**Results:**

Thematic analysis of interview transcripts revealed important benefits, concerns, and user requirements for smartphone-based HIV VL self-testing within the context of PLHIV lived experience, knowledge, and barriers to care in Indiana.

**Conclusion:**

End-user needs and preferences were identified as key design specifications and implementation considerations to facilitate the acceptability and inform ongoing development and ultimately real-world translation of the HIV VL monitoring device prototype.

## Introduction

HIV continues to affect approximately 38 million people globally [1], and over one million people in the USA [2]. With antiretroviral therapy and monitoring of this treatment efficacy, people living with HIV (PLHIV) can reach viral suppression, which not only enables them to live long and healthy lives but also prevents them from transmitting the virus to others [1,2]. Antiretroviral therapy reduces HIV viral load (VL) in the blood, with the goal of viral suppression (200 copies or less of HIV per milliliter of blood) [3] at which time the virus can no longer be transmitted to sexual partners [4–6]. Research suggests that PLHIV can reach viral suppression within 6 months of initiating therapy and can maintain viral suppression by adhering to their medication plan as prescribed [7]. However, only 66% confirmed cases of viral suppression were reported in 2019 [8], a far reach from the U.S. Department of Health and Human Services (HHS) Healthy People 2030 target to increase viral suppression among PLHIV to 95% [9] .

Indiana has some of the lowest percentages of PLHIV who received any HIV medical care (72% compared to 76% nationally), were retained in HIV care (48% vs. 58%), and who are virally suppressed (60% vs. 66%) [8]. Over 13,000 PLHIV were estimated living in Indiana in 2021, with disproportionately higher rates among Black and Hispanic individuals compared to White [10]. The HIV epidemic has coincided with increases in injection drug use, leading to an HIV outbreak in Indiana, affecting mainly rural communities with already limited health care resources [11].

Regular VL testing has the potential to facilitate medication adherence and safe behavior decision-making. However, current VL testing in the U.S. is based around a centralized healthcare system, involving laboratory-based platforms that can be expensive to maintain and time intensive due to the sample collection and processing techniques, equipment lab space, and trained staff required [12]. Clinical guidelines for VL testing published by the U.S. HHS can be as frequent as every 4 weeks for patients initiating or modifying antiretroviral therapy [13] and vary based on patient’s history of VL, CD4 count, and length of treatment.

The Healthy People 2030 HIV Working Group, the 2022–2025 National HIV/AIDS Strategy, and Ending the HIV Epidemic in the USA (EHE) are aligned initiatives aiming to end the HIV epidemic by 2030 and promote “finding innovative and effective ways to re-engage the estimated 250,000 individuals who are aware of their infection but not receiving HIV care and treatment” [14]. Leveraging new technology and point-of-care (POC) diagnostics for VL testing has the potential to reach those individuals by enabling self-, community-, or peer-based testing in a variety of settings (e.g., mobile clinics, convenient community locations, and home) and providing faster results to expand VL testing coverage and facilitate earlier access to antiretroviral therapy to improve viral suppression [15]. POC VL testing is already recommended by the World Health Organization as a method to monitor treatment among PLHIV receiving antiretroviral therapy [16]. Evidence suggests the cost-effectiveness, diagnostic accuracy, and feasibility of POC VL testing, as well as the potential benefits of increasing viral suppression, care retention, and improved quality of care and services. An emerging and growing area in HIV treatment, commercial near-patient tests are currently available [17] and more are in development [15]. A review of POC VL tests in development indicated that most tests have high specificity and sensitivity comparable to standard lab tests at detecting VL ≥ 1000 copies/mL [12], which is the WHO’s threshold for treatment failure [16]. While above the threshold for viral suppression defined by the U.S. HHS Guidelines, these tests are able to detect high levels of VL quickly to enable increased access to necessary treatment changes for the most vulnerable.

The ability to perform self-testing of HIV VL could enable individuals to more easily monitor their viral suppression status. However, current POC devices require trained professionals to collect blood and administer the tests, preventing their use in the home. To bridge this gap, smartphone-based diagnostic platforms [18] for VL testing have emerged with potential for home-based testing of HIV infection and progression [19–21]. One example of a handheld device that has been used for the testing of multiple pathogens and recently demonstrated to provide quantitative readout is the iSpy instrument at OmniVis Inc., which may be amenable to home use for HIV VL monitoring. Prototypes of iSpy leverage smartphone-based computation, communication, and imaging capabilities to quantify particle diffusion in response to amplification of pathogen nucleic acid gene targets. The detection capabilities have been demonstrated on *V. cholerae* bacteria in water samples, SARS-CoV-2 virus in saliva, and malaria-causing plasmodium parasites in blood [22–24]. Further, usability was evaluated with field technicians in Bangladesh to monitor water for *V. cholerae* contamination [25]. Quantitative HIV detection was recently demonstrated [26].

Such smartphone-based VL testing innovations have the potential to reach resource-limited communities and individuals most affected by HIV to address inequities in care. However, successful medical device innovation and translation requires meaningful investigation of end-user contexts and incorporation of those context-specific needs early in design processes [27]. Human-centered design approaches focus technology designers’ attentions on end-user needs, experiences, and contexts of use via prototype demonstration techniques and regular feedback loops with stakeholders throughout various stages of design processes. These approaches been advocated for use in global health applications due to their prioritization of stakeholders’ needs and lived experiences [28,29]. The objective of this study is to engage PLHIV and HIV healthcare providers in human-centered design research to inform key design and implementation considerations for a smartphone-based HIV VL self-testing device prototype. Specifically, we aimed to (1) identify benefits and concerns of smartphone-based HIV VL self-testing within the context of PLHIV lived experience, knowledge, and barriers to care in Indiana and (2) establish design specifications and implementation considerations that would facilitate the acceptability and real-world translation of the device.

## Methods

**Figure 1.**
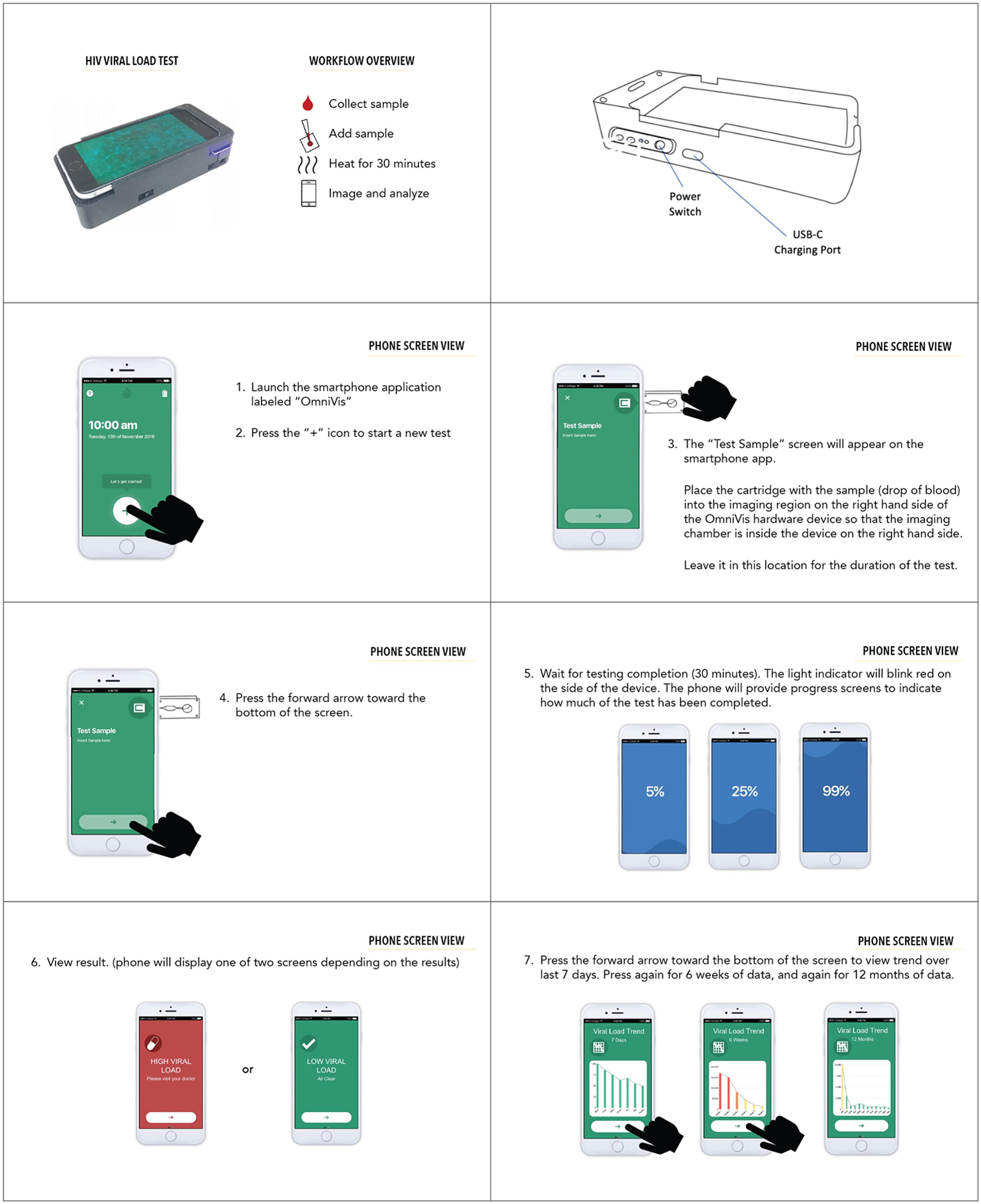
Digital prototype of smartphone-based HIV viral load self-testing device prototype shown to participants.

Semi-structured in-depth interviews were conducted with PLHIV and HIV providers. Interview guides were developed based on contextual investigation and human-centered design frameworks [27] in order to understand how this device may fit into the HIV care continuum in Indiana and to situate our findings on the acceptability of a smartphone VL testing device within the participant experiences and knowledge of HIV care. Questions were tailored to participant type (provider or PLHIV) and included demographic questions, open-ended questions regarding experiences providing or receiving HIV care, opinions on VL self-testing, a 7-item survey to assess VL knowledge and how VL knowledge influences behavioral intent (Table 1), and a digital prototype of the device displayed through PowerPoint demonstration (Fig. 1) followed by feedback-gathering questions on the device. An excerpt of the interview guide is included in the appendix.

**Table 1.** Survey questions

Scale of 1 (strongly disagree) to 5 (strongly agree)
If [I/a client] find[s] [my/their] level of infection (VL) is low or undetectable, it will encourage [me/them] to maintain the therapy
If [my/their] level of infection is low or undetectable, [I/they] will feel less worried
If I find [my/they] VL has gone up, [I/they] would be more likely to use condoms and practice safe sex
If [I/they] find my VL is going up, [I/they] am likely to go into the clinic for more testing or treatment
If [my/their] VL is undetectable, [I/they] can decide to have sex without condoms
When [I/people] know my VL, [I/they] [am/will be] better able to keep my sexual partner safer
[People understand that] A high VL means the risk of HIV transmission to my partner is higher

VL = viral load.

HIV providers were recruited via email through publicly available contact information provided on websites of HIV clinics throughout Indiana, and PLHIV were recruited through flyers posted at HIV organizations in Indiana (convenience sampling). Interviews were conducted by trained study team members via Zoom for approximately 60–90 minutes. Verbal consent was obtained from each participant prior to commencing interviews. After conclusion of each interview, each participant was sent a $50 electronic gift card.

All participants were assigned a pseudonym prior to data collection that are used in transcripts and reporting. Audio recording of the interviews was transcribed verbatim using Otter.ai, a digital transcribing platform, and then reviewed and edited by research assistants for accuracy.

Transcripts were thematically analyzed using a combination of inductive and deductive coding in NVivo, a qualitative coding software. An initial codebook was developed through deductive analysis of the interview guide, research questions, and preliminary review of transcripts, followed by open coding of all interviews to identify additional themes, and axial coding to review, synthesize, and categorize themes. Two independent coders coded each interview and met to reach consensus for each interview; any remaining discrepancies were resolved by the larger study team. Recruitment and interviews were concluded when it was determined that thematic saturation was reached by interviewers/coders recap of each interview in team meetings and report of no additional themes [30].

This study was approved by Purdue University’s Institutional Review Board (IRB-2021-1434).

## Results

### Contextual Investigation

A total of fourteen interviews were conducted with PLHIV (*n* = 10) and HIV providers (*n* = 4). Sixty percent of PLHIV participants were male, 60% were white, and the majority (70%) were employed and had health insurance (90%). The providers interviewed consisted of two physicians, one nurse practitioner, and one social worker. Years of experience working with HIV positive clients ranged from 7 to 20, with an average of 13.75.

**Figure 2.**
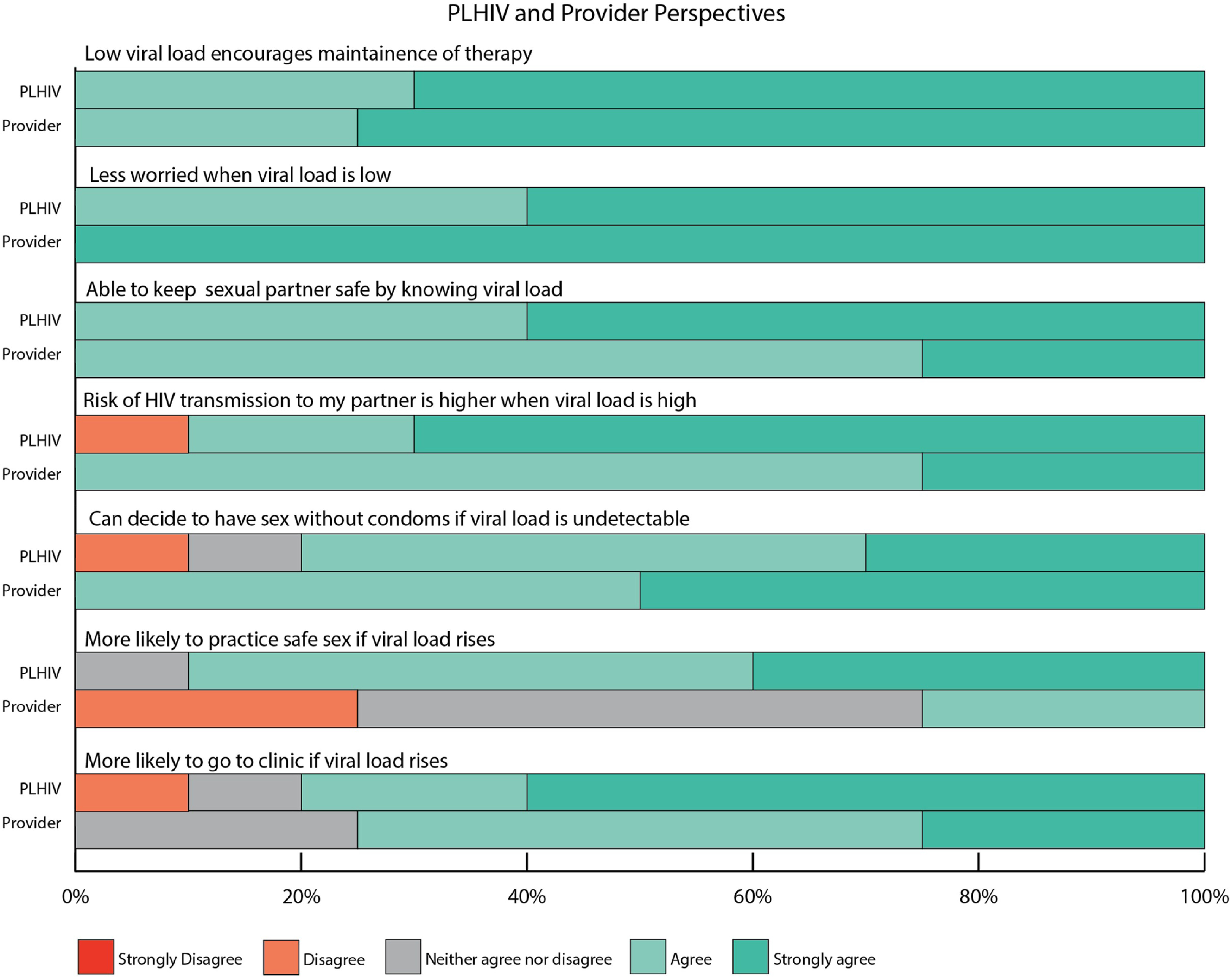
Viral load knowledge and behavioral intention (PLHIV = person living with HIV).

PLHIV were highly knowledgeable about VL and invested in knowing their own VL, citing it as *“essential”* to therapy management in terms of both adherence to medication and how well their medication was working. PLHIV were generally aware of any changes in VL over their course of living with HIV and which VL levels indicated undetectable or virally suppressed, *“I know less that 200 is generally regarded as like the mark point. And the way I think of VL suppression is once you’re regarded as undetectable, or they can’t find live copies of the virus in the sample, you are then unable to spread the virus via sexual transmission” (Barron, PLHIV).* Likewise, providers felt that in general most of their patients were knowledgeable about VL and invested in their status, “*I would say pretty much all of them know what a VL is. That’s kind of what they’re most concerned about. When we do labs and everything is, “am I undetectable? What is my VL”? Because they want to know that their risk of spreading HIV as partners and that sort of thing is minimal.” (Sarah, provider).* Patient self-report supports this; the majority of PLHIV (90%) knew that the risk of transmission is higher with a detectable or high VL (see Fig. 2).

Both PLHIV and providers reported that VL knowledge influenced attitudes and behavioral intent. All agreed that knowledge of having a low VL or undetectable status would encourage therapy maintenance and help feel less worried. However, providers reported more skepticism in terms of safe sex practices. While the majority of participants agreed that you can keep your partner safe by knowing your VL, only 25% of providers, compared to 80% of PLHIV, agreed that PLHIV were more likely to practice safe sex if VL rises.

In terms of participant experiences in HIV care, most PLHIV participants expressed going to a clinic twice a year for a blood draw and visit with their HIV provider, indicating successful disease management. Providers elaborated that the frequency of care varies patient to patient. *“It depends on how they’re doing clinically, at least twice a year. But for some individuals, it’s more frequently every four months … or even every two months if they are needing extra support or not doing well clinically.” (Brooke, provider)*

Though all PLHIV interviewed indicated high compliance with their care, they reflected on barriers experienced throughout the years since their diagnosis. These barriers included difficulties with access and navigating the health care system. Access to care was difficult at times for those who struggled with substance use, *“Before I got sober, I was not great about compliance” (Barron, PLHIV)* or were worried about COVID, *“The recent pandemic has made it more challenging, or at least [I] had some patients [that] didn’t want to come into medical facilities because they were nervous about being around others.” (Brooke, provider)*

In terms of navigating the healthcare system, scheduling conflicts were a common barrier. Even among those with high compliance, some participants said they missed appointments due to sickness or work, exacerbating existing challenges like long wait times and taking time off from work, *“My provider is excellent. But he has a waiting list of months so …scheduling can be difficult….” (Barron, PLHIV)*

Cost of testing also exacerbates the challenges with navigating and accessing care, *“I actually get my lab work done at my primary care doctor, because it’s free. It costs for me to get it done at the infectious disease doctor… when I know it’s time to get ready to see my infectious disease doctor, I’ll schedule an appointment at my primary care doctor to get the blood drawn… and then they send that information to my infectious disease doctor.” (Michelle, PLHIV)*

Providers also expressed frustration with the healthcare system, particularly when labs have to be done separately from appointments,

*“One of my Linkage-to-Care case managers… had been trying to get this guy into medical care. He kept rescheduling appointments. [He] finally goes, has his appointment. But the way that they do it at [clinic] is that you go see [doctor] and then you go down the hall and you get your labs drawn. So you wait twice, which isn’t like the end of the world. But the client totally left… In our [other region] their labs are always scheduled on a different day, so if they [patients] live in a different county, they have to come in for an appointment and then they have to come back in to get their labs drawn on a different day. There’s so many barriers to doing it … there’s a lot of people who right now aren’t getting their labs done that probably would if there wasn’t that barrier.” (Josephine, provider)*

These barriers, combined with high VL knowledge among PLHIV and their intent to mitigate risk behaviors based on their VL status, suggest that PLHIV would understand the meaning and what appropriate actions to take from self-tested VL results, and that self-testing potentially has a place in HIV care. Situated in this context, three key themes were identified regarding PLHIV and providers perspectives on smartphone-based VL self-testing: (1) perceived benefits and concerns of VL self-testing, (2) design considerations for future iterations of the device, and (3) implementation considerations for smartphone-based VL self-testing.

### Perceived Benefits and Concerns

Overall, PLHIV and providers were open to self-testing VL and liked the prototype,

*“I want to do that! This is making me excited just by looking at it” (Sadie, PLHIV)*

*“I think this would be relatively well-received by other HIV care providers. I personally think most of us like my age and younger would be very quick to adopt this. As long as the device itself is performed well.” (Adam, provider)*

**Figure 3.**
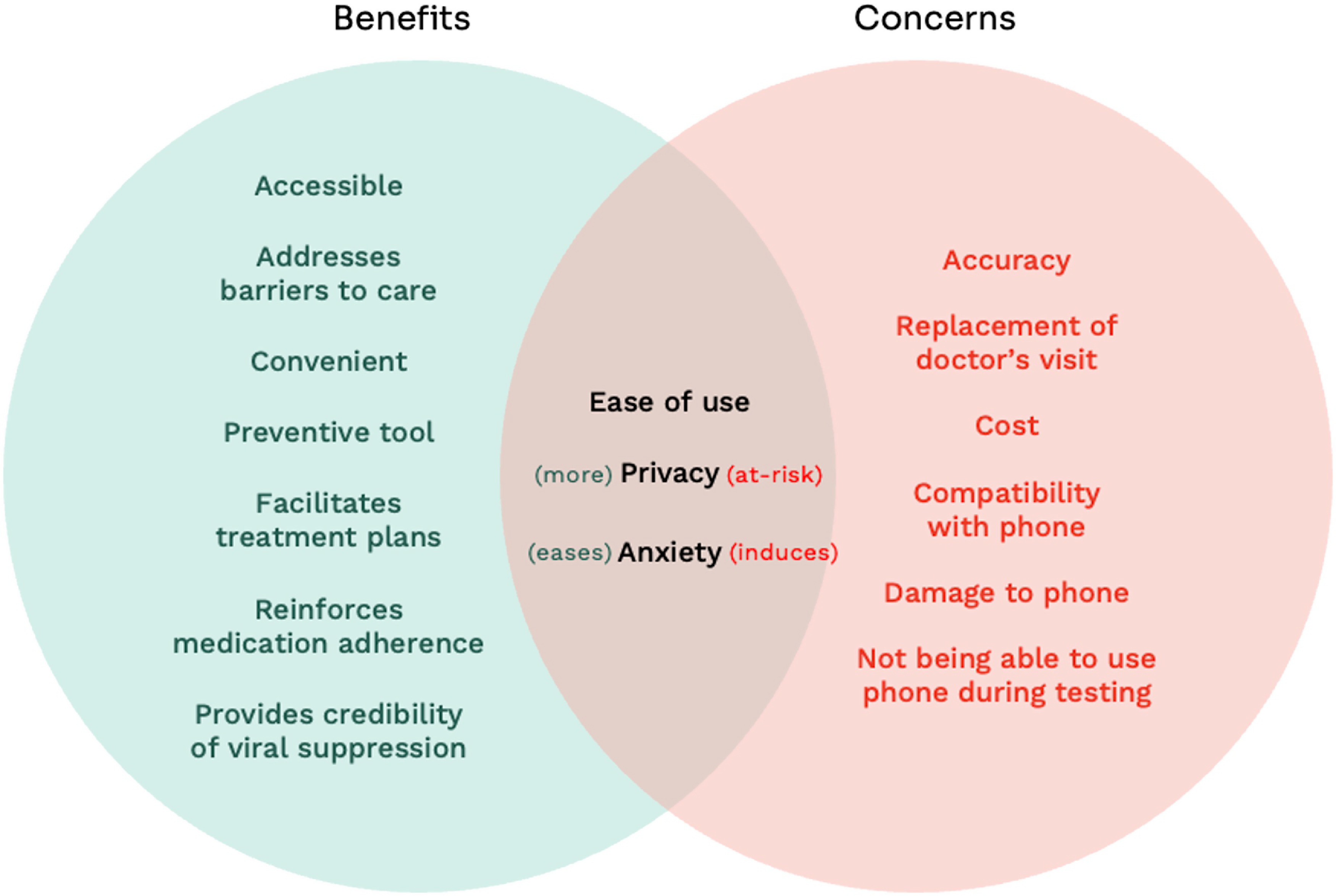
Benefits and concerns of smartphone-based HIV viral load self-testing.

As shown in Fig. 3, there were several benefits and concerns noted by participants, some of which overlapped. Being able to self-test on a smartphone would make VL monitoring accessible to all kinds of people, as Allan (PLHIV) suggests, *“Everyone’s got a phone… Everyone has the ability to test themselves.”*

The accessibility enables the potential to address key patient barriers, such as lack of transportation and insurance.

*“I think people being able to test at home would be a gamechanger for a lot of people…some people with HIV don’t get tested because they don’t have insurance, or they can’t get off work or they’d have to drive 100 miles.” (Barron, PLHIV)*

Even for those who do not face such barriers, the device is still viewed as convenient and for some even preferable than going to the lab *“I like the convenience… it offers a possible solution to having to physically go to a lab and have your blood drawn.” (Eli, PLHIV)*

Participants also suggested that VL self-testing can serve as an important preventive tool that can be particularly useful for certain populations or in certain scenarios (e.g., early in treatment before becoming undetectable, during medication changes),

*“For those people that work in the sex trade, this would be an invaluable resource. They can test themselves… when it comes down to it, we’re all trying to do the same thing and that’s rid the world of this terrible virus” (Donna, PLHIV).*

*“…if I switch a medication now to make sure my VL is still undetectable that would be useful” (Nolan, PLHIV).*

As Adam elaborates, it can facilitate treatment plans particularly for people early in treatment or at risk for treatment failure,

*“… for patients who if there’s a concern where there’s treatment failure… any difficulty because they’re not usually going to feel anything. So if they did send me an abnormal result… they’re like, “I’m really having trouble with getting my medications or substance abuse and other things,” that prevent them from being adherent, then I could get one like on the spot. So I think that’s the other part of it…well if you can’t come in right now, we can check your VL while you’re home, I’ll see where you’re sitting, and then we’ll go from there.” (Adam, provider)*

PLHIV also expressed how being able to know their VL as “*an instantaneous read, even if it is an hour” (Eli, PLHIV),* and viewing results over time on the app can reinforce medication adherence,

“*…if somebody who’s undergoing treatment can like visually see that graphic and the improvement.. those visuals help people remain compliant with medication, because it lets them see like, yeah, what’s actually working here, like visually in front of you.” (Barron, PLHIV).*

Additionally, it can provide credibility of viral suppression, *… if my partner wants to, like, have me prove that I’m suppressed, like I could do the tests and like show on the screen.” (Nolan, PLHIV)*

In terms of perceived concerns, accuracy of the VL test, and it replacing a doctor’s visit were mentioned. Providers, specifically, were concerned about VL self-testing interfering with necessary components of in-person care or even inconveniencing patients to come in for confirmatory or other testing.

*“… it would depend on how accurate and sensitive the testing was, and how it affected the patient’s engagement with overall care because that’s not the only thing that we need to check for them. We also want to check to make sure that we’re not causing any harm to their kidneys or liver or causing any issues with their white blood cell or red blood cell counts. So, if only one particular lab was being monitored remotely, it might not be as helpful if they also had to come in to get testing for all the other things. So, I think there’s definitely two different ways to look at it. In some respects, it could be very helpful” (Brooke, provider).*

Cost and compatibility with phones were also concerns, particularly for PLHIV,

“*…daily testing would be super cool, but I don’t know how like expensive that could wind up getting in, though (Barron, PLHIV).”*

*“Not all the phones still get the same type of connectors in the same spot as you listed there… Don’t get me wrong. This sounds neat. I like this idea. I really do. But it’s also making it so it’s compatible with other phones.” (Jaime, PLHIV)*

One PLHIV questioned if the device would cause damage to their phone, “*I also would be interested in… how would it affect my phone like … you said it would heat up the sample like would that heat go in my phone… How would that affect my phone… would it overheat… that kind of thing?” (Nolan, PLHIV).* Other PLHIV emphasized how integral phones are to daily lives and how not being able to use phone while the test is running may not be ideal, “*Here’s the real question is when someone will not use their phone for 30 minutes?” (Eli, PLHIV).* Despite this, some PLHIV expressed how they would incorporate testing without phone use into their routine, *“What I would do would be to time those tests. That is later in the evening when I’m not expecting phone calls. And I just tried to use my noggin my common sense and do it at a nonbusy time of the day.” (Donna, PLHIV)*

Ease of use and privacy of the device, as well as anxiety from self-testing VL, were themes that presented as both perceived benefit and perceived concern. For ease of use, most participants found the device to be simple, straightforward, easy to use, and portable (benefit),

*“I like that it seems simple and straightforward. And anybody that has basic knowledge of a smartphone could use it.” (Jamal, PLHIV)*

*“It’s small enough, it’s mobile, you can take it with you wherever you go.” (Josephine, provider)*

While some acknowledged that the device may be difficult for certain people (concern), others felt that using the device operation can be learned (benefit), similar to learning other home monitoring tests like glucose.

*“I think there’d be some who they’re just not great adopters of technology in general…. I just think our older generations would still be struggling with the virtual visits. So pricking their finger and putting them on a phone, you know, unless they have something else like diabetes or something, you know that they’re used to using little devices at home to measure blood sugar or other things. It’s going to be a challenge.” (Adam, provider)*

*“Yeah, it seems pretty self-explanatory. I mean, likening it back to glucose monitoring. We have people with all different levels of education and health literacy that can be taught how to how to monitor their blood sugar, and it’s very simple. It seems like a very simple walk you through the process sort of app and that’s really helpful.” (Brooke, provider)*

Most PLHIV also felt that self-testing VL would ease anxiety (benefit) by providing peace of mind,

*“If something went wrong with taking my medication if I missed a couple doses or if I have some other issue that I think might be affecting my VL like it would give me some peace of mind.” (Nolan, PLHIV)*

However, providers thought that VL self-testing could also potentially induce anxiety (concern) by patients testing too frequently and misinterpreting fluctuations in VL readings that are not clinically meaningful,

*“I think that could really ease somebody’s mind and make them feel better, but at the same time, there were a lot of people who would just like literally be testing all the time and getting anxiety over “oh my gosh, 30 today. Oh my gosh, 15 today. Oh my gosh, you know, why am I not undetectable?” So there’s power in knowledge, but sometimes it’s overkill” (Sarah, provider)*

In terms of privacy, Allan (PLHIV) felt that self-testing VL provided more privacy (benefit) *“…it’s added privacy because my port is home. You know what I mean?”* whereas Jamal (PLHIV) expressed the risk of data breach as a threat to privacy (concern), “*the biggest one [concern] is just knowing that things like this can get breached, the privacy part.”* However, for Michelle (PLHIV), the technological advancement of a self-testing VL device is worth the possibility of data breach, which is an inherent risk in digital storage of medical information practiced today, *“…. nothing is just really private at all… it’s no different really, than it being in the system in the computer system, or a database, which can be breached anyway…”*

Few PLHIV were not concerned with privacy of VL or HIV status, “*I pretty much announce those with a megaphone these days because I’ve been undetectable for quite a number of years now…” (Donna, PLHIV).* However, many PLHIV and providers stressed the importance of VL and HIV status, and that unconsented access to data can have serious consequences ranging from discrimination to criminalization.

*“it’s absolutely imperative to keep my VL private… even if I spit on somebody you know, I can be charged with a felony… if they were able to access what my VL was on a particular day to incriminate then, no, you do not [want them to] have access to that information.” (Eli, PLHIV)*

*“…we’re very, very protective of our patient’s privacy… because we know the implications of their HIV status being revealed in a way that’s out of their control… we’ve had lots of people who have been discriminated against for employment or for housing or for other things because their HIV status was learned.” (Brooke, provider)*

Participants perceived benefits and concerns highlight smartphone-based VL self-testing as an attractive and desirable option for HIV care, particularly for those experiencing barriers to traditional care, and informed design and adoption considerations for future iterations.

### Design Considerations for Device Prototype

Design considerations were conceptualized as what technology developers need to know to design the next iteration(s) of the device. The design considerations listed in Table 2 came from participant feedback on aspects of the device they liked and ways in which it could be improved. These included accompaniments to the device, features/changes to the physical device, features/changes to the app design, privacy and data sharing requirements, and additional information to include in the app.

**Table 2.** Design considerations for device prototype

Theme	Subtheme	Quotes/Examples
Accompaniments	Finger prick kit	*“It would be cool if it came like in a little pouch where like your lancets can go in there and your little slides that you have to stick it- like so that everything could be kept together. That would be helpful.” (Josephine, Provider)*
	Device instructions in a variety of formations (e.g., video, written)	*“We all learn in different ways. So I think there should be options for all three of them. Some people are visual, some people have to be taught, some people like reading.” (Jamal, PLHIV)*
Assay	Lower limits of detection	*“Even though some studies used over 400 are currently [suppressed] over 200 we [at the clinic] would say you’re not, that’s not considered suppressed. That’s definitely a reason to talk to your doctor. I think between 200 and whatever the threshold for undetectable is that is chosen if it’s less than 30 or less than 20. I think that would still be a reason to talk to your doctor because there’s some, somethings going on that’s keeping that individual from having an undetectable VL.” (Brooke, Provider)*
Physical device	Charging port for phone	*“Would I be able to charge my phone while it is heating?” (Aja, PLHIV)*
	Compatibility with different models/sizes of phones	*“… lots of people have lots of different types of smartphones would it only be able to work with certain ones, and that that may limit some, some people’s ability to, to even access it because people have all different kinds of phones?” (Brooke, provider)*
Privacy Protection	Password protection and biometric controls (e.g., fingerprint/facial recognition)	*“I really like when they’re able to use that like biometric control.” (Barron, PLHIV)*
	Discrete app name and icon	*“I would also have the option to make the, like, icon, like on their home screen or whatever, something very nondescript, something that doesn’t point to it being an HIV tracking thing. And that will kind of be my other worry, is people sort of knowing what the icon looks like even if it is something nondescript. You know, that the name has nothing to do with HIV.” (Sarah, Provider)*
	Control over how to store data and for how long (e.g., locally on device or in cloud)	*“Some people might want to store you know, five years of data on their phone and show ‘look I’ve been undetectable for five years’ sort of thing. Some might not want that, so I think if there was an option, you know, where they could just check a box or you know, slide something on the screen that’s like keep records for a year, keep records for five years, store to the cloud, you know, keep the most recent 10 records. I think they should all be sent to the clinic and then we would have them in indefinitely. So I think that’s something that the patient should be able to decide” (Sarah, Provider)*
Sharing Information with Medical Providers	Automatically share medical results with providers	*“If there’s a way that that data could be, you could select to have it automatically sent to your provider… the auto information to your provider, I think would be an excellent feature.” (Barron, PLHIV)*
	Include specifics of data as well as additional information (e.g., date of test, VL result, if they took their medication, how they are feeling, and a notes section)	*“It would be really interesting if you could capture a couple of things… like whether or not they were ill at the times. So, if someone is sick with an upper respiratory infection, or gastroenteritis or something, their VL may get all out of whack… it would be really interesting to also get a sense of their adherence to their antiretroviral regimen in the time preceding any VL testing.” (Brooke, Provider)*
	Frequency of data report to providers	*“if it was someone who was needing, for some reason, needing to test very frequently, I wouldn’t want to wait a whole year before understanding where their VL was. If it was someone who’s just monitoring a couple of times a year, then maybe, they got to that point because they were more stable, and we wouldn’t need to see it as frequently.” (Brooke, Provider)*
App Design	Simple interface	*“I like to keep things really simple because when things are complicated for me, I shut down.” (Michelle, PLHIV)*
	Value neutral colors for results	*“But high VL isn’t wrong. So why is it red?… Red is cautionary right?” (Eli, PLHIV)* *“Wait. Wait, something’s wrong. Code Red, right.” (Eli, PLHIV)*
	Language that is familiar to PLHIV (e.g., detectable/ undetectable instead of high/low VL)	*“I would go with like the detectable/undetectable. That’s kind of what’s been conditioned into patients just because it’s the way our assays readout.” (Adam, Provider)* *“For me suppressed is a lot blurrier whereas detectable versus undetectable is fairly black and white. So, I prefer those terms.” (Barron, PLHIV)*
	Numerical data	*“I definitely wouldn’t use high and low and I wouldn’t use all clear either. I literally say you know, suppressed- um If it was suppressed or undetectable, I wouldn’t do anything besides like great. It’s green… and if it was detectable or unsuppressed I would say like, please contact your doctor, but I would have the number displayed. So… high VL like what does that mean? Is it 21 when our threshold is 20 or is it, you know, 200,000? That’s a big difference.” (Sarah, Provider)*
	Reminder/trackers (e.g., medication, appointment, and/or symptoms)	*“There’s still some clients who need to be reminded about taking pills right now, so a little bit of help with would be a good idea on that perspective.” (Jaime, PLHIV)*
	View of data over time	*“I think if the data were stored. Like, all results were stored in the app, like in a way that you could just like review them over time. That would be cool too, because then you could like, not only know your data points, but you could see any trends that might be occurring” (Barron, PLHIV).* *“I like being able to look at trends over time” (Adam, Provider)*
	Progress indicator to indicate test completion or error	*“I love that because I think we all need that feedback that something is actually working.” (Josephine, Provider)* *“It would be nice if like a could like sending an error message if something is not being done correctly. To remind to remind the person that oh, you, you forgot a step or something like that.” (Nolan, PLHIV)*
	A menu of preferences to customize features and privacy settings	*“Something that would be cool. Yeah, like when you log in and like, you know, add whatever demographics you want to, like, often would you like to test like, chime and be like, hey, you know? Test day or whatever. That’d be cool.” (Barron, PLHIV)*
Additional information	HIV information and resources	*“Just basic what is VL, what is a CD4 count…Just sort of basic education, what is u equals u?” (Sarah, Provider)* *“I think that having some sort of information on how to be a self-advocate or even have resources to help advocates would be valuable” (Eli, PLHIV)*
	Inspirational messaging	*“I think content about positive affirmations. Uh just reminders, calmness… so things like tricks on how to take medication. If you can’t swallow the pills or if you miss a dose, it’s okay, you know those types of things that you would normally get from a support group that can automatically be on the device would be great.” (Michelle, PLHIV)* *“I mean they could go so far as to put in little positive sayings. For you everyday to look at or go pick me ups.” (Donna, PLHIV)*
	Tailored messaging	*“Yeah. Yeah. Something cute and sweet on their birthday. Just to let em know you’re thinking of them and give them a reason to be happy that they made it through another year swallowing those great pills [sarcasm]” (Donna, PLHIV)* *“Like if your VL is undetectable, or you have a low VL or whatever it states that maybe you get like, some sort of affirmation, like you’re doing great or keep up the keep up the good work” (Eli, PLHIV)* *“If there is educational message messaging, that would be result dependent. So, if their VL was higher, it’s just reminding them like, ‘hey, you know, you’re more likely to transmit to someone else with this.’” (Adam, provider)*

PLHIV = person living with HIV; VL = viral load.

### Implementation Considerations

Implementation considerations were conceptualized as what public health practitioners need to know to implement the next iteration(s) of the device in real-world settings (Table 3).

**Table 3.** Implementation considerations for smartphone-based VL self-testing

Theme	Subtheme	Quotes/Examples
1. End user(s) / setting	Determine user and use case through shared decision making between patient and provider	*“… it’s really interesting to be able to have someone just check in and see how their medicines are working, especially if they’re unable to get into clinic or… maybe like in someone who’s restarted on medicines, and they want to see if their VL’s coming down… I think it would have to be kind of a shared decision making between the provider and the patient on whether or not this type of monitoring would be right for them.” (Brooke, provider)*
	Offer device as a POC option at provider’s office	*“… going to get labs was a barrier where if you could do it… in the office… it’s just so much more accessible to people.” (Josephine, Provider)*
2. Testing frequency	Place limits on testing frequency (user shouldn’t test too frequently)	*“it’s great to empower people with a certain perspective, we got to place limits on it. I don’t see that happening with this, but it’s more like, you know, it’s it lets people, you know, test at home, communicate directly with physicians, but But yeah, you just have to play some limits on on how patients use it.” (Adam, provider)* *“… it would be almost more like a PRN or an as needed thing in my mind. Like, here I’m writing you this order for so many self tests per year and you can use those at your discretion like for maybe four tests a year, six tests a year you know, if you feel like you need to check that, but it’s not a replacement for coming to your medical visits.” (Sarah, provider)*
3. Cost	Obtain insurance coverage and low cost for patients (PLHIV participants reported willingness to pay for $0-$100 as a co-pay for the device)	*“… if it was something that was free, I would, I would use it.” (Nolan, PLHIV)* *“Depending on the price point and if I could get it through my insurance, yeah, I definitely would [pay for it]. Ideally, if I could get it through my insurance and have a small copay for it” (Eli, PLHIV)*
	Use additional methods to obtain funding	*“How much? How much is this device going to cost? How will it be distributed? You know, will the cost of the device be taken care of by NASO or Ryan White or Medicare, Medicaid all of that,” (Eli, PLHIV)*
4. Privacy	Leverage current security technologies in healthcare	*“It has the same security measures as anything else. And we have applications for like, our bank accounts and credit cards and all that. So like this I mean, it’s health. You know, it’s health information. But we already have, you know, I have an app that accesses my EMR so I can pull up patient’s charts through that. That’s all secure by the same, you know, face ID and encryption and things.” (Adam, provider)*
5. Data reporting	Consider how/what self-testing data to report to state/national surveillance groups	*“Like that needs to get reported, like it needs to come back. Those results need to go to their provider, and then they need to go to the state because we’re looking at like community viral suppression across the state and ending the epidemic.” (Josephine, provider)*
6. Partnerships	Partner with Health Departments and provider to rollout	*“… working really closely with, with providers of HIV care, even local health departments would be would be something to do to kind of help market this as a public health intervention.” (Brooke, provider)*
	Associate with reliable institution(s) to facilitate trust in device	*“And then also like the app tied to some sort of institution that like I trust. For instance, if it was tied to university, I would trust it more than like, a private company or something… If it was tied to a hospital, I would trust that… But if it’s just like some sort of, I don’tdo not know, company I’ve never heard of or. I would trust it less.” (Nolan, PLHIV)*
7. User-testing	Conduct more user testing with handheld prototype	*“… like a prototype, like being able to come back and say, “Hey, this is what we developed. What about it works, it doesn’t work for you.” (Adam, provider)*

PLHIV = person living with HIV; VL = viral load.

## Discussion

This study employed a human-centered design approach to explore the acceptability and key user requirements for a smartphone-based HIV VL self-test prototype among PLHIV and HIV providers. Our findings reveal key stakeholder perspectives around the benefits and concerns of such a technology in the USA, and specifically Indiana. Others have explored the acceptability of HIV VL self-testing in the UK [31] and South African contexts [32]. Our findings support the overall acceptability and benefits reported in these studies including convenience and improved accessibility, while contributing additional, context-specific considerations, concerns, and preferences.

While self-testing for VL is not yet a reality, emerging technologies such as the one described herein are currently in development and enthusiasm for such innovations has been made evident by leaders of the ending the HIV epidemic (EHE) initiative [33]. In the USA, federal funders including the Point of Care Technology Research Network under the National Institute of Biomedical Imaging and Bioengineering are soliciting proposals to further advance HIV VL detection technologies, in order to address ongoing needs [34]. Such technological innovation must be accompanied by meaningful consideration of intended end-users of the technology as well as other key stakeholders [27]. This study provides a model for human-centered approaches to health technology design in ways that incorporate end-user feedback early in the design process, which can ultimately facilitate and improve clinical translation [35].

Stakeholder interviews provided key insights and considerations to inform both the design and future implementation of the proposed HIV VL self-test. While many of these user requirements are immediately actionable by technology developers (e.g., test accompaniments and app design suggestions), others provide important guidance that must continue to inform ongoing technology development, including results readouts, VL limits of detection, and smartphone compatibility. Participants highlighted the importance of providing more precise numerical readouts for VL measures or the use of thresholds “detectable” versus “undetectable” rather than broad ranges or interpretations such as “high” and “low.” This design specification would be similar to self-monitoring of blood glucose (SMBG) using fingerprick blood samples. While accuracy of SMBG devices approved by the FDA is ± 15% [36], these readers provide precise numerical readouts rather than a range of the result’s confidence interval. Indications of “High” or “Low” are only given when outside of the range of the SMBG meter’s calibration. It is reasonable to expect that POC HIV VL monitoring devices would also provide specific numerical readouts while maintaining a broader range of confidence intervals, although no standards currently exist and should be developed.

With higher limits of detection in POC or self-tests, current research and perspectives on self-testing for VL have discussed this concern regarding lower sensitivities in comparisons to laboratory tests [37]. Studies of POC VL detection have targeted 1000 copies/mL, the World Health Organization threshold for treatment failure [12,38] in order to achieve high sensitivity compared to centralized laboratory-based assays. However, as laboratory detection has become more sensitive, previously undetectable very low level viremia (below 200 copies/mL) was at the forefront of at least one participants’ (provider) concerns for patient monitoring. It is unlikely that VL self-tests would be able to achieve limits of detection below 200 copies/mL given the smaller blood sample volumes that are collected from fingerprick samples compared to venous blood draws. A recent WHO policy brief defines “suppressed” VL as detectable but ≤ 1000 copies/mL and states that PLHIV who have a suppressed but detectable VL and on medication as prescribed have almost zero or negligible risk of transmitting HIV to their sexual partner(s) [39]. The indications for use of a self-test for HIV VL will need to be carefully defined, whether for virologic treatment failure or for undetectable treatment as prevention, without overstating capabilities.

Finally, the use of a smartphone-based device led to interpretations that test developers may not consider when focused on the detection and assay alone. PLHIV assumed that the smartphone in use would be their own device and indicated concerns about interruption to their daily life and about accessibility and equity of the self-test. Concerns about equity and accessibility have been expressed previously as well [31]. Privacy concerns and suggestions extended beyond the device and discrete app design and into the connectivity to the providers’ EHRs.

Regarding implementation considerations, key insights highlighted the importance of established partnerships with local health departments, as well as concerns about patient interpretation of results and potential for over-testing. Major initiatives like EHE have established structures for partnership development, including with healthcare providers, Ryan White clinics and health centers, health departments, and others to expand capacity, strengthen systems, establish new programs and services, and to tailor and implement new approaches as appropriate in their communities [14]. Additionally, throughout the interviews, providers tended to express more concern for patient interpretation of results and the provider–patient relationship than patients did. In our study, providers worried about patient over-testing and the possibility that patients may fail to understand fluctuations of VL in response to stress and illness, while PLHIV did not express this reservation. Neither group was certain that PLHIV would see their provider in response to increases in VL. This could be encouraged with app design indicating that a patient should check in with their provider when VL increase was detected or when PLHIV felt ill.

Ongoing efforts will focus on adapting the current device prototype to incorporate these user requirements as design specifications and to continue to acquire iterative feedback from a larger and more diverse group of potential end-users. While this majority-White participant group is reflective of the population of Indiana (over 75% White, 9% Black, and 8% Hispanic) [40], the prevalence of HIV disproportionately impacts communities of color (46% White, 39% Black, and 10% Hispanic)[10], thus warranting oversampling in these populations. Furthermore, a key limitation of this study is that our recruitment strategy relied on flyers posted at HIV organizations and thus all PLHIV participants were already connected to care. Future work will aim to reach vulnerable communities in which PLHIV may not yet be connected to care as well as engage broader stakeholders, including health departments, policymakers, and payers, on device usability and feasibility in intended settings, to ensure the successful translation and implementation of the technology.

## Supporting information

Rodriguez et al. supplementary material Rodriguez et al. supplementary material

## Supplementary material

The supplementary material for this article can be found at https://doi.org/10.1017/cts.2023.686.
