# Supplementary material for: Human-centered design of a smartphone-based self-test for HIV viral load monitoring
Source: J Clin Transl Sci. 2023 Nov 24;7(1):e262. doi: 10.1017/cts.2023.686 (PMC10790236; doi:10.1017/cts.2023.686)
Supplement: Rodriguez et al. supplementary material [file S2059866123006866sup001.zip › supplementary material (2).docx]

## **SUPPLEMENTARY MATERIALS**

**Supplementary Table 1. Interview Guide Excerpts**

##### ***Same question asked to provider and PLHIV**

| Providers | **PLHIV** |
| --- | --- |
| *Provider experiences with HIV care and opinions about HIV VL self-testing*  How many clients with HIV does your clinic care for each year?  How many do you care for directly?  How often do your meet with your clients regarding their HIV care?  *Do your clients at your clinic know what an HIV VL is and what causes their VL to change?  Do you think they understand the risks of having a high VL?  What VL threshold, or other method, do you use to determine if a client’s treatment regimen needs to be changed?  Do you or someone from your clinic inform clients of their VL testing results? If so, what information do you provide them about their VL? If not, why not?  How often do you need to change treatment regimens for your clients?  *Would it be useful for patients to be able to test and monitor their VL on their own? Why or why not? How would that information be useful?  What possible problems could this solve? What possible problems could this cause?  *If clients were able to find out their own VL, how often should they test themselves?  *If clients tested themselves for their VL, should they keep their own records? (yes/no/not sure)  *Where or how would you want them to keep them?  *Should clients report the VL test results to their provider on a regular basis? (Every time/Only when it changes/Not sure)  *If it were possible to have an automatic report sent to your clinic every time the client performs the self-tests, would that be a good idea? (yes/no/not sure). Why/why not? | *PLHIV experiences with HIV care and opinions about HIV/VL self-testing*  When were you initially diagnosed/when did you learn your HIV status?  Are you currently seeing a clinician or care provider for your HIV care? How often do you meet with your provider or care provider regarding your HIV care?  How do you schedule appointments with your provider/provider? (schedule follow-up immediately after an appointment in person, follow up email/phone, online, etc.)  Have you ever missed an appointment?  If so, why? [barriers]  *Do you know what an HIV VL is and what causes your VL to change?  Do you know what your VL is now? (If yes) Do you know how much your VL varies?  How do you find out the level of your VL?  Do you think it is useful for a person living with HIV to know what their VL is? (yes/no/not sure). (If yes) How is that information useful?  *If you could test your VL on your own, do you think you would want to test it? Why or why not? If not, who is the best person to test VL?  *If you had a way to find out your own VL, how often would you like to test yourself?  *If you could test yourself, would you like to keep your own records? (yes/no/not sure)  *Where or how would you want to keep them?  *Would you want to report the test results to your provider every time or only if there is a change? (Every time/Only when it changes/Not sure)  *If it were possible to have an automatic report sent to your clinic every time you test, would that be a good idea? (yes/no/not sure). Why/why not? |
| *Provider and PLHIV perception of the HIV VL smartphone model and its use, after viewing*  What things do you like about this model and app?  Do you think it would be easy for [you/your patients] to operate this model? (yes/no/not sure)  What concerns would you have about [your patients] using this model and/or the app?  VL readout: is high/low ok? Or would you prefer to see High/med/low? Suppressed/unsuppressed? Or do you need to know # viral copies? Or just relative over time? (show examples)  Does this smartphone-based device look like it would be easy to carry and store? What makes the device seem easy or not-so-easy?  Doing a medical test usually requires some sort of test strip or kit that has to be thrown away after use. If it is just a small strip or chip, similar to what diabetics use for testing blood sugar, would [you/your clients] be able to dispose of it easily? (yes/no/not sure)  What do you think is the best way to learn how to use this HIV VL smartphone model? (Individual demonstration/on-line video/written instructions/other ideas)  Are there other aspects of the phone application that might be helpful [for your patient’s care]? Some examples might be medication reminders, appointment reminders, or messages with other supportive information? If so, how could this help?  Do you have other advice or questions for the designers of this HIV VL smartphone device that could make it better to use? | |
